# ‘It’s the same thing as giving them CPR training’: rural first responders’ perspectives on naloxone

**DOI:** 10.1186/s12954-022-00688-4

**Published:** 2022-10-03

**Authors:** Matthew R. Filteau, Brandn Green, Frances Kim, Ki-Ai McBride

**Affiliations:** 1 JG Research and Evaluation, Bozeman, MT USA; 2 Montana Department of Health and Human Services, Behavioral Health and Disabilities Disorders Division, Helena, MT USA

**Keywords:** Naloxone, Layperson Training and Administration, Harm Reduction, Frontier and Remote (FAR)

## Abstract

The Substance Abuse and Mental Health Services Administration’s (SAMHSA) Harm Reduction grant program expanded access to several harm reduction strategies to mitigate opioid overdose fatalities, including expanding access to naloxone. Interviews with first responders in a frontier and remote (FAR) state were conducted to understand their job responsibilities in relation to overdose response and prevention and their perceptions of training laypersons to administer naloxone. This study includes 22 interviews with law enforcement, EMS and/or fire personnel, and members of harm reduction-focused community organizations. The study finds widespread support for increasing access to naloxone and training laypersons in naloxone administration throughout Montana, due to rural first responders’ inability to meet the needs of residents and an overall lack of resources to address addiction and the effects of fentanyl. Participants from harm reduction-focused community organizations convey support for training lay persons, but also illuminate that real and perceived cultural opposition to harm reduction strategies could reduce the likelihood that laypeople enroll in naloxone training. This study adds to the literature because it focuses on first responders in a FAR area that would benefit from layperson naloxone education and administration training due to its geographic expansiveness and the area’s overall lack of access to medications for opioid use disorder or other treatment services. Expanding harm reduction approaches, like increasing access and training laypersons to administer naloxone, might be FAR residents’ best chance for surviving an opioid overdose.

## Introduction

Opioid overdose (OD) deaths in the USA continue to rise, and approximately 68,630 Americans died in 2020 from an opioid-involved overdose [53]. The US Department of Health and Human Services has declared the rise in opioid use and deaths an epidemic and national public health emergency [23]. The Substance Abuse and Mental Health Services Administration’s (SAMHSA) Harm Reduction grant program expanded access to several harm reduction strategies to mitigate overdoses. The funds allocated resources to community harm reduction services and promoted access to “... sterile syringes, safe sex kits, prevention education about synthetic opioids and other substances, overdose prevention kits including naloxone distribution, peer worker engagement, medical services, case management and referral to treatment” [66].

These recent community harm reduction provisions complement previous efforts to expand naloxone (common brand name Narcan) across law enforcement officers (LEOs) and emergency medical technicians (EMTs)^1^ [47]. In 2013, the US National Drug Control Policy center urged all law enforcement agencies to carry naloxone because they often respond to overdose calls before EMTs arrive [9]. This is particularly relevant in rural areas where an absence of advanced-level EMS providers, who are more likely to administer naloxone, contribute to the higher rate of opioid-related overdose deaths and lower rate of naloxone administration by emergency medical services [26, 82]. Opioid overdoses require an urgent response and quick naloxone administration times, which are prolonged when EMTs misperceive that they themselves may overdose when encountering fentanyl. Winograd et al. [80] discovered that trainings mitigated EMTs’ fears about overdosing from incidental contact with fentanyl. Other studies document that informational naloxone trainings increase administration competency and decrease EMTs’ apprehensions about administering naloxone [82], while Kilwein et al. [39] demonstrate that basic life support personnel felt confident managing an opioid overdose even before they received training on how to respond.

Another factor that may impede first responders’ effectiveness is a reluctance to call emergency medical services among people who use opioids (PWUOs) when confronted with a peer experiencing an overdose. PWUOs are more often to be present during an overdose but stigma and legal repercussions [22, 43, 61, 74, 78] decrease their willingness to call for help. To alleviate this hesitancy, states enacted Good Samaritan Laws (GSLs) to provide overdose victims and witnesses immunity from prosecution for the possession of controlled substances and/or drug paraphernalia when they report an overdose in good faith [21, 41]. However, despite the good intentions of GSLs and some positive effects from these laws, laypeople and people who use opioids were initially hesitant to call 911 fearing repercussions [8, 20, 75]. With time, GSLs increased engagement among first responders and reduced the rates of fatal OD [14, 49, 56, 62]. The potential reluctance to call first responders and wait times prompted calls for laypeople and PWUOs to receive naloxone to reduce administration time and increase administration [55].

Peers of people at risk of an opioid overdose have a better chance than first responders to immediately administer naloxone due to proximity to the individual experiencing an overdose [37]. Recent studies find that PWUOs demonstrate high levels of naloxone administration competency [55, 37], and opioid education and naloxone distribution programs that provide take-home naloxone rescue kits (NRKs) reduce opioid overdose death rates [16, 55, 77].

Structural barriers such as inaccessibility and a lack of knowledge about where to obtain naloxone, particularly for rural residents [25, 72], and the stigma ascribed to PWUOs deter people from obtaining naloxone [5, 70]. Rural residents confront additional barriers because some pharmacists are unable or unwilling to stock naloxone, and rural residents who do obtain it have been found to receive less comprehensive administration instructions than urban residents [72]. As Albert et al. [1] demonstrates, most PWUOs fall into a gray area between low and high-dose opioid prescription (≥ 100 mg/day morphine equivalence) use, and Bailey and Wermeling [4] find that people prescribed high doses of opioids are more receptive to naloxone education than people who use opioids illicitly. Other factors motivate lay people to support take-home NRKs, such as knowing someone who experienced a fatal or non-fatal overdose [78] and being politically liberal [11].

This study draws on Bessen et al.’s [7] conclusion that multiple points of naloxone access in communities, especially rural communities, decrease fatal overdoses. The present study examines first responders’ perceptions of take-home NRKs for laypeople in Montana, a state with numerous Frontier and Remote (FAR) areas. This study adds to the literature by examining first responders’ perceptions of expanding naloxone access to laypeople in a state with FAR areas that lack MOUD treatment and mental health care options [2, 34, 64].

## Methods

### Study context

This study was part of a broader evaluation of the State Opioid Response (SOR) grant program from the Substance Abuse and Mental Health Services Administration (SAMHSA) as administered in the state of Montana. The larger study sought to understand first responders’ perceptions of MOUD treatment programs and harm reduction measures, particularly about naloxone distribution and use within Montana. Montana’s Behavioral Health and Developmental Disabilities Division (BHDD) of the Department of Public Health and Human Services identified first responders as key stakeholders, and naloxone training and administration as the special topical area for research. The study was submitted to Western IRB for approval and received an exempt status (Approval #: 13093595).

The state of Montana has had a relatively low overdose mortality rate during the opioid overdose crisis as compared to other states. During the study period (2020), the Opioid Poisoning Age-adjusted Death Rate in Montana was 7.3 per 100,000 residents, compared to a national rate of 21.4 per 100,000 [24]. This rate in 2020 rate was a marked increase from 2017 to 2018 in Montana (2.4 per 100,000), which lead BHDD staff to concentrate on the expansion of harm reduction strategies, including naloxone distribution via a pharmacy standing order, per the state medical officer. The standing order was used as a strategy to increase access to naloxone by reducing the barriers to making a request for the medication, with funding for naloxone being provided by the SOR grant funding. In this context, the study was intended to support BHDD in identifying barriers to receipt and administration of naloxone to reverse an overdose among first responders.

### Sampling

The project team compiled contact information for: (1) naloxone trainers and master trainers,^2^ (2) law enforcement agencies, and (3) EMS service providers from all 56 counties in Montana. The sampling frame prioritized counties that received naloxone via a standing order with the state and that had a population above 1000 people—40 out of 56 counties in Montana. Recruitment occurred via email and over the telephone; however, because much of rural Montana relies on volunteer EMS and fire departments, no formal staffing lists exist, which presented challenges for our tele-recruitment efforts. This resulted in an overrepresentation of police officers in the sample, as they are paid, identifiable, and accessible by phone and email.

### Data collection

The final study sample included 22 interview participants: eleven law enforcement officers, eight EMS and/or fire personnel, and three members of community organizations dedicated to harm reduction in Montana. Data collection ended once saturation was reached: when the information gleaned no longer provided new themes or theoretical relevance to emergent harm reduction insights [18, 31]. One senior member of the research team conducted all the interviews, which averaged approximately 45 min. The interviews for this study focused on understanding naloxone use, distribution, and administration training. The interviews were audio recorded, transcribed verbatim and coded by two members of the research team [18]. To ensure coding reliability, two coders resolved discrepancies through a “negative case analysis,” whereby researchers refined the working hypothesis in the context of negative and disconfirming evidence to ensure all patterns fit the study’s conclusion [3, 50].

**Fig. 1:**
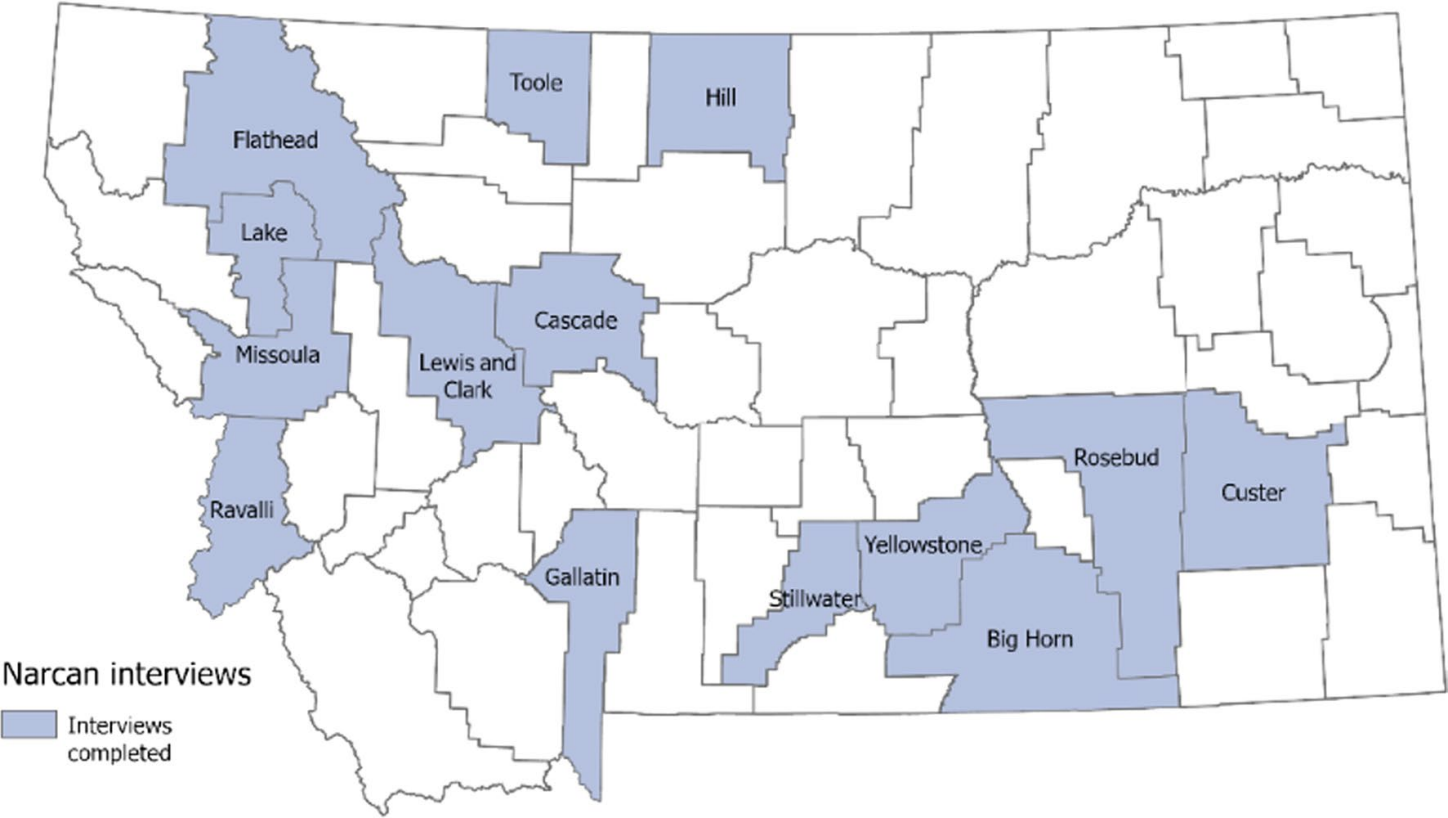
Map of study participants

Figure 1 shows the counties where respondents were located. Montana is a predominantly rural state, as defined by the US Department of Agriculture Rural–Urban Commuting Areas. RUCA commuting areas were used to classify the census tract, as they reflect a rural–urban gradient based upon population density, urbanization, and daily commuting. Each of these patterns may affect overdose response patterns by first responders (Table 1).

**Table 1:** RUCA designation of study participants

County in sample	RUCA designation
Missoula	Metropolitan
Cascade, Yellowstone	Micropolitan
Lewis & Clark, Gallatin, Big Horn	Small town with commuting
Flathead, Toole, Hill, Ravalli, Custer	Small town little commuting
Lake, Stillwater, Rosebud	Rural

### Analysis

Initial coding allowed the research team to “remain open to all possible theoretical directions indicated by [...] the data” [15: 46]. Through initial coding, the coders identified “Challenges” as a prominent pattern facing first responders who administer naloxone in Montana. Then, during a second stage of focused coding, coders identified “Distance” and “Lack of Resources” and “Perceived Opposition to Harm Reduction” as three salient categories that explain the challenges first responders faced in Montana and the difficulties training laypersons to administer naloxone [67]. The research team then coded Distance for the sub-category “lack of first responders.” We also coded Lack of Resources for the sub-categories “lack of emergency medical service providers” and “lack of treatment options.” We then coded Perceived Opposition to Harm Reduction for “stigma.” Through focused coding, we were able to understand how the challenges for first responders systematically interrelated as facilitators and barriers to training laypeople to administer naloxone [17: 55, 67].

## Results

### Distance

The distance first responders must travel to service calls and to administer naloxone emerged as the most consistent theme among interview participants in both Montana’s more and less populated counties. For example, one rural EMS provider states: “Distance. Yeah. We are such a rural county; we have one ambulance station, and we cover about 2000 square miles. So, time and distance to be able to get to a patient is sometimes very difficult.” This pattern was present among all types of first responders; for example, a county deputy recounts: “Law enforcement, our agency, we are very far from a lot of our calls. It's not uncommon for us to have a 30-min, 40-min runtime to our location. We are, by far, the quickest and fastest unit, but we're spread out.” Distance in a frontier and remote setting presents other challenges for first responders, such as which calls to prioritize:

I would say probably for us, obviously distance. We are the sole ambulance for the county and it's like 12,000 people and almost 4,000 square miles. Depending on the road, if you get off the pavement even on nice high grade county roads, it might take us two or three hours to reach the edge of our county, depending on how you're getting there. [. . . ] Often the helicopter coming out of Billings can beat us. [. . .] I would say for overdoses, it’s the access to law enforcement. We don’t have that much law enforcement and we don’t have enough staff and they’re so busy and often understaffed that they’re not truly able to go to every call like that before us and clear all of those.

This participant describes a vast service area that an emergency helicopter out of Billings can cover quicker than ‘local’ first responders. In addition, to manage limited resources, participants shared that dispatch rank the most urgent calls, ensuring that responses meet the most immediate needs while accounting for distance and travel time to service in alignment with common 911 dispatch practices [68].

First responders in Montana’s most densely populated areas were not exempt from difficult routes and long travel times for service. For instance, one metropolitan EMS provider recounts: “And just because of the highways and the distances in Montana, we respond to some really faraway places that are probably… We call them ‘dead zones’ where there's just not a lot of responders available.” Based on our interviews with first responders, much of Montana could be classified as a ‘dead zone’: a geographically isolated area with a persistent lack of services. Further complicating their job, EMS in Montana are assigned large coverage areas that strain their modest crews. One rural EMS participant states: “On our normal shifts we have a four-man crew, but sometimes faced with vacation and comp and that kind of stuff. We may have a three-man crew and running a three-man crew on an unresponsive [overdose] patient … you need more people.” First responders in Montana report covering large swaths of the state, often understaffed.

### Lack of resources

**Fig. 2:**
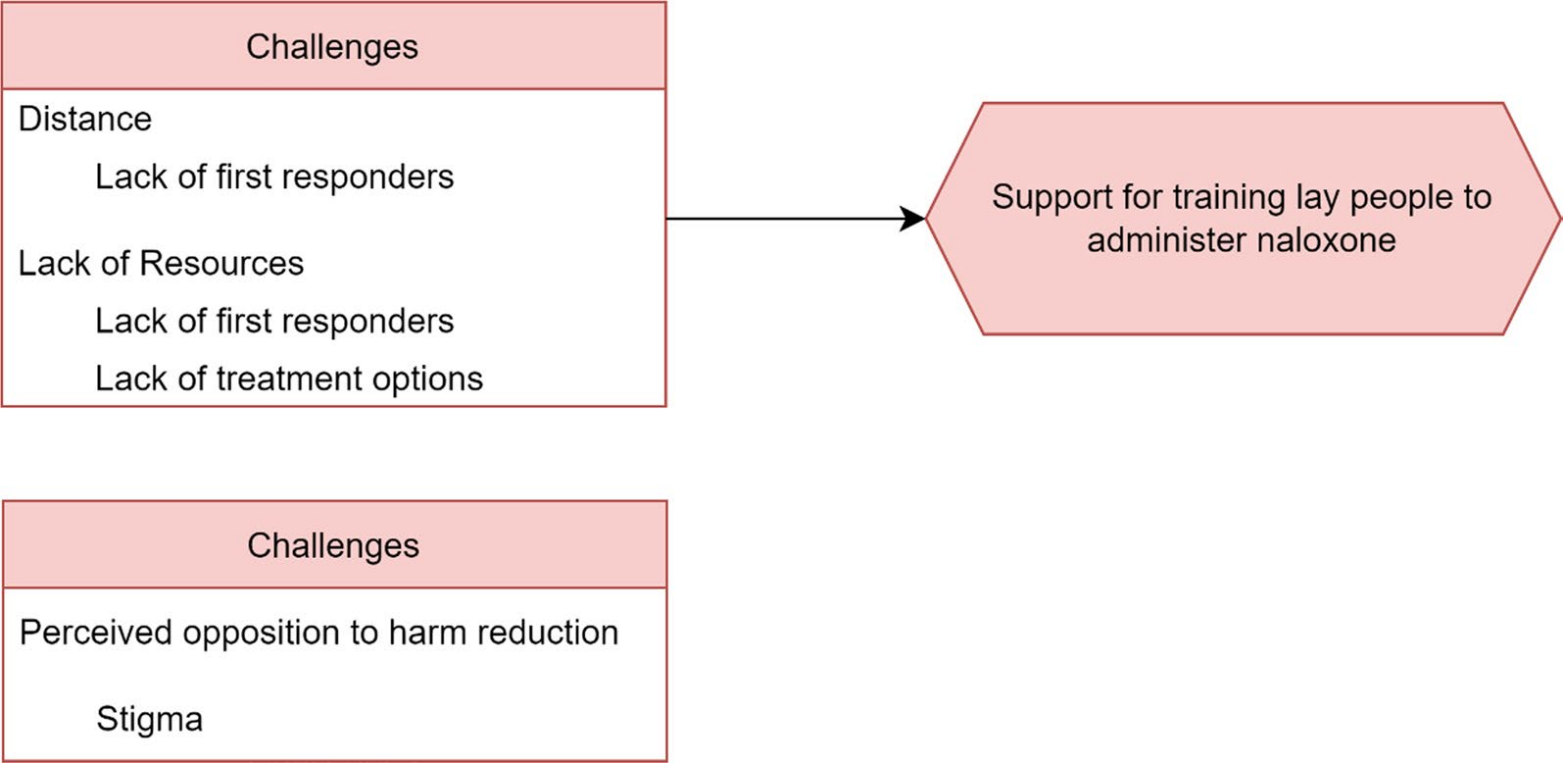
Coding structure

The large service coverage areas that first responders patrol interrelates with the overall lack of available resources across Montana’s vast landscape. For instance, Montana lacks treatment and recovery programs in rural areas which forces residents to travel long distances for services. One rural county’s EMS director stated: “Access to substance abuse treatment and mental health…I mean, we don't have resources here. The only behavioral health unit close by is often full.” Montana’s frontier and remote counties lack vital services and rely on those offered in Montana’s, often distant, more densely populated areas. Figure 2 displays the number of MOUD providers by county in Montana, noting that 42 of Montana’s 56 counties have 2 or fewer waivered providers for prescribing Buprenorphine. A law enforcement officer in one rural county notes:

I don't know if you could ever have enough [treatment] resources. But being a small community where there's not a ton of resources, I know the clinic has kind of a MOUD program. We do have drug court here through district court that the sheriff sits on. So we try to do things, but like AA has fallen off a lot. You don't see the AA meetings or the NA meetings as much. I think we went from three or four meetings every week to having trouble finding one meeting in our area every week. So stuff like that's fallen off. I think just when you're in a smaller community, there's just not ever going to be enough resources to get people in to, and they're going to have to travel to bigger areas. Like for us, it would be Great Falls or Kalispell where there's going to be more resources available.

Participants in rural counties rely on urban centers that have more available resources, but those resources are often unable to handle the demand within and across counties. A law enforcement officer in a metropolitan county states:

You could interview officers daily who, somebody is on drugs and they want to get treatment right now. And we all know that if you have an addict who needs treatment or wants it, right now, when they're willing is the best thing in the world. And our guys will call up there and they're like, "Yeah, we don't have any beds." And so, we got nowhere to take them.

A firefighter working in one of Montana’s micropolitan counties recounts:

We have a lack of social services in [Name] County. To include what you're asking, also mental health, those prevention programs and things like that, I don't think we're a big enough community yet to have all the resources that we need, and that's going to continue to be a bigger and bigger problem as the county grows as fast as it is, and we see it all the time in different scenarios, not just drug abuse, but mental health is another one. Our geriatric patients or our older patients, we just lack social services, and our homeless community, there aren't a lot of resources, and we don't even have a homeless shelter that's open year-round. We don't have a homeless shelter that's open 24 hours a day. I mean, we're just not there yet. There's a lot of services that we need that we just don't have yet. So, I guess just as a citizen, I would say no. There's probably not enough resources for drug abusers.

Montana’s first responders, regardless of their service area’s population density, describe facing the same challenges: a lack of mental health and substance use disorder treatment resources for their residents. The dearth of services contributes to first responders’ support for training laypersons in naloxone administration.

### Community training

This study’s interviewer asked first responders whether they supported or opposed training community members in naloxone administration. Most participants supported training every willing participant, while some supported training certain community members, and only one participant opposed training and providing naloxone to community members. A rural law enforcement officer states: “I think it would have to be selective. Some providers, that would benefit from the Narcan training [would be] mental health people that we have here in town because that's, like I said before, it kind of coincides with some mental health issues.” An EMS director in another rural county agreed: “When we have a person that we've identified as being at risk of overdose, having some key people that are frequently around that patient or person, I think [the targeted training] would be the best use of resources.” Some respondents felt training individuals closest to those at risk of overdosing was the most efficient way to ensure naloxone was within reach of an overdose victim; however, other participants questioned this tactic, especially in rural areas where community members would be unwilling to identify as PWUOs or an individual with high-risk contacts. One rural law enforcement officer captures this sentiment: “I just don't think that there would be a big turnout for the people that would be the most at risk because they wouldn't want to put themselves out there.” This participant alludes to the stigma associated with identifying oneself as high-risk in rural communities in Montana, suggesting that the targeted approach recommended by previous participants might not work in some areas.

To circumvent the challenges with training select community members, respondents viewed the pandemic, and the distances first responders need to cover as a justification for training laypersons who lack a direct connection to PWUOs. A member of the Montana Department of Justice states:

[Narcan] should just be available to all walks of life because... Not only to people that can afford it since... But all the way down to people that are struggling, that they could have an opportunity to be administered or given Narcan so that they could use it because they have family members and loved ones that could be and are addicted to opioids. And it could be lifesaving for them at some point as well.

The previous respondent expressed support for training laypeople Montana, a rural state where first responders face vast coverage areas and lengthy response times. Other first responders who support training community members equated naloxone administration training to other forms of first aid trainings, such as CPR and AED training. For example, one rural law enforcement officer states: “It never hurts. It's the same thing as giving them CPR training, AED training. You hope they never have to use it, but if they did, at least they'd be confident in using it.” An urban firefighter echoed the previous participant: “I teach CPR classes [to the] general public. My opinion on the matter is yes, and whether it's stop-the-bleed classes, or CPR, or Naloxone, or anything like that. I think there's always a benefit there.” The distances first responders cover in Montana and the lack of resources within communities affects participants’ attitudes toward training and administering naloxone among laypersons. Rural Montanans suffer from a more severe lack of access to resources; however, participants in this study largely regarded all of Montana as underserved and supported layperson naloxone administration training throughout the state.

Support for training community members also emanated from a place of concern about the amount of substance use and a perceived opposition to harm reduction in Montana. A member of Montana’s harm reduction community stated, “Now we give naloxone to everyone and we actually educate meth users to just be aware of fentanyl [and that it’s in] meth. So, it doesn't really matter what substance people are using, we do the education with everyone.” Fentanyl’s pervasiveness and the likelihood people who use drugs encounter a lethal dose compels members of the harm reduction community to support training lay people to administer naloxone. Another member of Montana’s harm reduction community advocated for training laypersons because,

Well, I think it's better to do it than not. [. . .] People try to keep what they do here pretty secret, because we've got a lot of fundamentalists. The [County] is pretty conservative. People are not open to talking about use. They're not understanding, they're pretty judgmental. [. . .] So on one hand, yes. I think that again, providing that education through Naloxone training would be important, but I also don't know if people are as open about use as they are in other places. So while I would hope that the community would be open to learning about it, I don't know how effective it would be.

Harm reduction advocates who participated in the study describe that stigma exists throughout Montana, and this sentiment may prevent people in key roles from completing naloxone administration training. The previous participant fears that real and perceived cultural opposition to potentially beneficial harm reduction strategies within her community could reduce the likelihood that laypeople enroll in naloxone training.

## Discussion

Rural communities experience a 45% higher rate of opioid-related overdose deaths than urban areas, less access to knowledge about opioids, and fewer evidence-based harm reduction services [25, 26, 66, 82]. Some FAR states constrain effective harm reduction strategies: Montana’s Board of Medical Examiners still requires EMRs and EMT-Bs to get an extra “Naloxone Endorsement” training to administer naloxone, restricting naloxone availability [52]. Participants in the current study largely echo previous studies that suggest providing laypersons with access to naloxone training and administration can mitigate overdoses [37]. Members of harm reduction-focused organizations questioned whether community members would be willing to obtain naloxone due to cultural opposition to harm reduction strategies.

This study has implications for harm reduction community groups and practitioners because it examines take-home NRKs for laypeople and the challenges facing first responders in a FAR state with limited treatment and harm reduction services. Montana residents have limited access to needle exchanges, and persistently low recruitment and retention rates for peer support specialists [27, 36]. First responders in this study respond to these shortcomings by describing a lack of access to treatment and harm reduction services throughout Montana. Structural barriers constrain Montana’s ability to expand medication for opioid use disorder (see [34]), prompting calls for a more robust harm reduction strategy that compensates for the state’s insufficient treatment capacity [12]. One method that could reduce these structural barriers and improve layperson access to naloxone is the implementation of a mail-order naloxone program [81].

National data from 2018 showed that too few (only 42%) substance use treatment providers offered medication for opioid use disorder (US Substance Abuse and Mental Health Services Administration, 2019), and many treatment options were concentrated in urban areas, which resulted in long wait times for admission and an increased risk of overdose for rural patients [2, 34, 57, 64]. Previous studies suggest that a stronger connection to treatment and recovery services can bolster harm reduction efforts [71, 76, 37]; however, first responders located in the most remote and developed areas of Montana reported serving residents across vast geographic distances and expressed frustration with the lack of addiction treatment resources and lengthy waitlists that limit access to treatment for Montanans (also see [13, 30, 60]). Expanding harm reduction approaches, like training laypersons to administer naloxone, residents’ best chance to reverse and survive an opioid overdose due to the persistent lack of treatment services in FAR states [2, 34].

## Limitations and future research

The findings should be interpreted while considering this study’s limitations. First, this study utilized a convenience sample to generate emergent, logical inference, rather than produce statistical generalizability [73]. In addition, the availability of law enforcement officers and difficulty recruiting rural, volunteer fire and EMS crews via phone and email led to an overrepresentation of LEOs in the sample. Montana is a unique study location, and although many states lack FAR areas, future research should continue to study the implementation of harm reduction strategies in politically conservative FAR states like Montana, because people in areas who lean right of center politically often oppose harm reduction strategies [11], and argue that harm reduction promotes rather, than deters, addiction (see [7]).

Montana’s conservative context likely affects the colloquial language first responders use, and additional research should examine whether stigmatizing language used by first responders, that contrasts person-first language, affects the implementation of harm reduction strategies in FAR areas. An explicit focus on the implications of this stigmatizing language on the adoption of practices was beyond the scope of this research study; however, understanding the barriers that exist for and the policy suggestions from people who use drugs in politically conservative FAR states would illuminate how these participants would meet their needs. Researchers should also study how harm reduction strategies like the expansion of peer support affect opioid use and overdose rates, especially in states like Montana with a history of low retention and trouble recruiting qualified peers [27]. Lastly, research should examine how stigma ascribed to PWUO from first responders interacts with structural barriers that prevent PWUO from accessing treatment in FAR areas [12, 22, 29, 32, 35, 43, 61, 69, 74, 78].

## Conclusion

States are actively expanding access to harm reduction strategies to mitigate the opioid overdose epidemic [66], and many FAR areas are predisposed to barriers that inhibit the implementation of these services. Understanding first responders’ views of take-home NRK in FAR areas has implications for harm reduction community groups and practitioners by illuminating the barriers and gaps facing residents in FAR areas. Expanding access to naloxone and other harm reduction strategies becomes essential, especially in areas where severe structural barriers prevent residents from accessing harm reduction services and treatment.

## Data Availability

The datasets generated and analyzed for the current study are not publicly available. JG Research and Montana’s Department of Public Health and Human Services share ownership and are available from the corresponding author upon reasonable request.

## Footnotes

1 We use “first responders” interchangeably when referring to emergency medical technicians (EMTs), firefighters, and law enforcement officers (LEOs).

2 One organization was awarded a contract by the state of Montana to train first responders and lay people to become Naloxone trainers: individuals qualified to train first responders (master trainers), and laypeople (trainers) to administer Naloxone.
